# Breaking of the Trade-Off Principle between Computational Universality and Efficiency by Asynchronous Updating

**DOI:** 10.3390/e22091049

**Published:** 2020-09-19

**Authors:** Yukio-Pegio Gunji, Daisuke Uragami

**Affiliations:** 1 Department of Intermedia, Art and Science, School of Fundamental Science and Technology, Waseda University, 3-4-1, Ohkubo, Shinjuku, Tokyo 169-8555, Japan; 2 Department of Mathematical Information Engineering, College of Industrial Technology, Nihon University, 1-2-1, Izumi-cho, Narashino, Chiba 275-8575, Japan; uragami.daisuke@nihon-u.ac.jp

**Keywords:** cellular automata, trade-off, computational universality, computational efficiency, asynchronous updating

## Abstract

Although natural and bioinspired computing has developed significantly, the relationship between the computational universality and efficiency beyond the Turing machine has not been studied in detail. Here, we investigate how asynchronous updating can contribute to the universal and efficient computation in cellular automata (CA). First, we define the computational universality and efficiency in CA and show that there is a trade-off relation between the universality and efficiency in CA implemented in synchronous updating. Second, we introduce asynchronous updating in CA and show that asynchronous updating can break the trade-off found in synchronous updating. Our finding spells out the significance of asynchronous updating or the timing of computation in robust and efficient computation.

## 1. Introduction

Michael Conrad, who explored biocomputing based on a protein chip, described how molecular interactions can implement computation by regarding the conformation changes in molecules as the state changes in the computation [1,2]. If any two molecules with different conformations colliding with each other rapidly lead to one specific conformation, then the computational efficiency is very high, although the computational universality is very low. In contrast, if two molecules colliding entail one molecule whose conformation can be constantly modified, it implies that the various states of computation can be accessed by these molecules and that the computational universality is very high. Since some conformations arrive after the long wandering of conformation changes, the time to access these conformations is so long that the computational efficiency is very low. This thinking results in the trade-off principle between the computational universality and efficiency in bioinspired or natural computing [1]. After Conrad, although various biomaterial computing techniques have been developed while referring to that trade-off, the relation between natural computing and the trade-off is still unclear since computing is usually based on the Turing machine [3,4,5,6,7,8].

The trade-off principle is ubiquitously found in biological systems as the dilemma between generalists and specialists [9,10,11,12,13]. If the environment in which a species lives is constantly changing, and if the species has not adapted to any specific environment too much, then the species can live in the various environments to some extent. This species is called a generalist. In contrast, if a species is adapted only to a specific environment, the species is called a specialist [10,11]. The contrast between a specialist and generalist is also found in machine learning. An excessive generalist is compared to undercomputing in learning, while an excessive specialist is compared to overfitting in machine learning [14].

While the trade-off and/or the dilemma suggests that the computational universality (generalist) and efficiency (specialist) can be quantified and compared with each other, they are neither systematically argued nor quantified in a certain space of the computation. Instead, the contrast between the universality and efficiency might be compared to the phase transition between chaos and order [15,16,17,18,19]. The chaotic dynamics implementing state wandering can be compared to a universal and low efficiency computation, and the oscillating dynamics can be compared to non-universal and high efficiency computation. The specific dynamics implementing state wandering among multiple attractors can be compared to the critical state or balance of the universal and highly efficient computation and is sometimes called the edge of chaos or self-organized criticality [20,21,22]. In the phase transition, the chaos and order can be quantified with respect to the order parameter corresponding to the temperature. The phase transition is found not only in the continuous dynamics but also in cellular automata (CA) [15,23,24] by using the chaos and order in their behaviors [25,26,27].

Although the edge of chaos in CA suggests balancing the universal and highly efficient computation, there is little research to bridge the phase transition with the trade-off between the universality and efficiency. On the one hand, the computational universality has been strictly investigated in terms of the Turing machine [28,29,30] and/or logical gates [31,32]. For instance, it has been argued as to how to implement a universal Turing machine as simply as possible in CA [27,29]. In this framework, the machine either has universality or not, and there is no notion such as the degree of computational universality. On the other hand, there is no strict research on the relation between the edge of chaos and the balance of the universality and efficiency. Although the computation at the edge of chaos might contribute to balancing the universality and efficiency, it has not been determined how the critical computations are close to the optimal solution and/or balancing. Thus, self-organized criticality is used not as the search for optimal solutions but as metaheuristics [33,34]. Because there is no quantification to bridge the universality and efficiency, no detailed research proceeds on this issue. Therefore, it is a novel idea to quantify the degree of universality and bridge the computational universality and computational efficiency.

It is remarkable whether the perspective of the phase transition between chaos and order is founded under the framework of synchronous updating. Rather, there is no synchronous clock in natural biological systems or biocomputing, and they work by asynchronous updating. This behavior implies that asynchronous CA can emulate natural computing in the sense of Conrad’s research. If the transition rule is asynchronously updated to cells, then behaviors such as the critical state are ubiquitously found in the rule space of CA and are referred to as the universal criticality [35,36]. Recently, asynchronous updating in CA has been shown to lead to the phase transition coupled with the critical state expressed by the power law [37,38,39,40]. While knowledge regarding the large difference between synchronous and asynchronous updating has accumulated [41,42,43,44,45,46], there has been little research on how asynchronous updating in CA can influence the trade-off between the universality and efficiency and/or the phase transition of chaos and order.

With this background, first, we define the computational universality and efficiency in the behaviors of elementary cellular automata (ECA) and quantify the degree of the universality and efficiency. We show that there is a trade-off between the computational universality and efficiency in synchronous ECA. This is the first attempt to elucidate the trade-off between the universality and efficiency in the research field of CA. Second, we show that the asynchronous updating in ECA can break the trade-off principle and analyze what contributes to the break of the trade-off. This work provides a novel perspective on how asynchronous updating can play a role in balancing universal and efficient computing.

## 2. The Trade-Off Principle in Synchronous ECA

Since ECA was proposed by Wolfram, some rules have been studied by information processing and by constructing logical gates [25,26,27]. Most of them are studied in the form of synchronous updating. ECAs are defined by a set of the binary sequences of cells, **B***^n^* with **B** = {0, 1} and a transition rule *f_r_*: **B**^3^ → **B**, where *f_r_* is synchronously updated to all cells and *r* represents the rule number mentioned below. The transition rule with synchronous updating is expressed as

*a_i_^t^*^+1^ = *f_r_*(*a_i_*_−1_*^t^*, *a_i_^t^*, *a_i+_*_1_*^t^*). (1)

If a transition rule *f_r_* is adapted to all cells in **B***^n^* (i.e., global adaption), then we assign the global use by *G*(*f_r_*): **B***^n^*^+2^ → **B***^n^* such that

(*a*_1_*^t^*^+1^, *a*_2_*^t^*^+1^, …, *a_n_^t^*^+1^) = *G*(*f_r_*) (*a*_0_*^t^*, *a*_1_*^t^*, …, *a_n_*_+1_*^t^*). (2)

The transition rule is coded by the rule number, *r*, such that for *x, y, z* ∈ **B**,

*s* = 4*x* + 2*y* + *z* (3)

*d_s_* = *f_r_* (*x, y, z*) (4)

*r* = Σ^7^*_s_*_=0_2*^s^d_s_*. (5)

The rule number, *r* = 18, is represented as R18, where *d*_1_ = *d*_4_ = 1 and *d_s_* = 0 with *s* ≠ 1, 4. There are 256 rules in ECA since there are 2 possible outputs for 8 inputs of a triplet.

How can one define the computational universality and efficiency? Given an initial state of **B***^n^* with random boundary conditions, reachable states are determined by a transition rule. For the case of R0, only one state consists of all 0 for any initial states; this implies that (0, 0, …, 0) = *G*(*f*_0_)(*a*_0_, *a*_1_, …, *a_n_*_+1_) for any (*a*_0_, *a*_1_, …, *a_n_*_+1_) ∈ **B***^n^*^+2^. By contrast, R204, of which *d*_2_ = *d*_3_ = *d*_6_ = *d*_7_ = 1 and *d*_0_ = *d*_1_ = *d*_4_ = *d*_5_ = 0, can show that (*a*_1_, *a*_2_, …, *a_n_*) = *G*(*f*_204_)(*a*_0_, *a*_1_, …, *a_n_*_+1_) for any (*a*_0_, *a*_1_, …, *a_n_*_+1_) ∈ **B***^n^*^+2^ and that all possible states can be reached if an adequate initial condition is prepared. It is easy to see that R204 shows a locally frozen pattern (class 2). For R90 or R150, all possible states can be reached, although the generated patterns are chaotic (class 3). Thus, the ratio of reachable states for all possible initial conditions can reveal the computational universality. Given 2*^n^* all possible initial states with random boundary conditions, the computational universality of rule *r*, *U*(*r*), is defined by

*S*_R_(*r*) = {*G*(*f^T_r_^*)(*a*_0_, *a*_1_, …, *a_n_*_+1_) ∈ **B***^n^* | (*a*_1_, …, *a_n_*) ∈ **B***^n^*, (*a*_0_, *a_n_*_+1_) ∈ **R**(**B**^2^)} (6)

*U*(*r*) = #*S*_R_(*r*) (7)

*U*_N_(*r*) = *U*(*r*)/2*^n^* (8)

where for a set *S*, #*S* represents the cardinality of a set *S*, **R**(**B**^2^) represents one element set randomly determined from **B**^2^, and superscript *T* represents *T* numbers iteration of *f_r_*. If *n* = 2, then *U*(0) = #{(0, 0)} = 1, and *U*(204) = #{(0, 0), (0, 1), (1, 0), (1, 1)} = 4 *U*_N_(*r*) represents the normalized computational universality. Here, we call elements of a set, *S*_R_(*r*), reachable states.

Next, we define the computational efficiency of a transition rule *r*. To separate from the computational universality, the computational efficiency is expressed by the average time to reach the reachable states. For each reachable state *X* ∈ *S*_R_(*r*), the average time to reach *X* represented by τ*_r_*(*X*) is expressed as

(9) $$\tau_{r}\left(X\right)=\underset{Y\in B^{*}}{\sum }T\left(G\left({f^{T}}_{r}\right)\left(Y\right)=X\right)$$

where B* = **B***^n^* × **R**(**B**^2^), *T*(*G*(*f^T^_r_*)(*Y*) = *X*) implies time *T* such that *G*(*f^T^_r_*)(*Y*) = *X*. Since the time *T* is computed for any *Y* ∈ B*, it can lead to *G*(*f^T^_r_*)(*Y*) ≠ *X*. At that case, if *G*(*f^T^_r_*)(*Y*) = *X* is not obtained within 2*^n^* time steps, then *T*(*G*(*f^T^_r_*)(*Y*) = *X*) is a constant value, *T_θ_*. For the case of R204 in which any initial condition is not changed by the transition, *G*(*f_r_*)(*Y*) = *Y* with *T* = 1 and then for any *X* ∈ *S*_R_(*r*), τ*_r_*(*X*) = (1 + *T_θ_*(#B* − 1)). The computational efficiency is defined by

(10) $$E\left(r\right)=\underset{X\in S_{R}\left(r\right)}{\sum }\tau_{r}\left(X\right)/\#S_{R}\left(r\right)$$

Since *E*(*r*) is the average time to reach the reachable state, the smaller *E*(*r*) is, the more efficient ECA *r* is.

**Figure 1:**
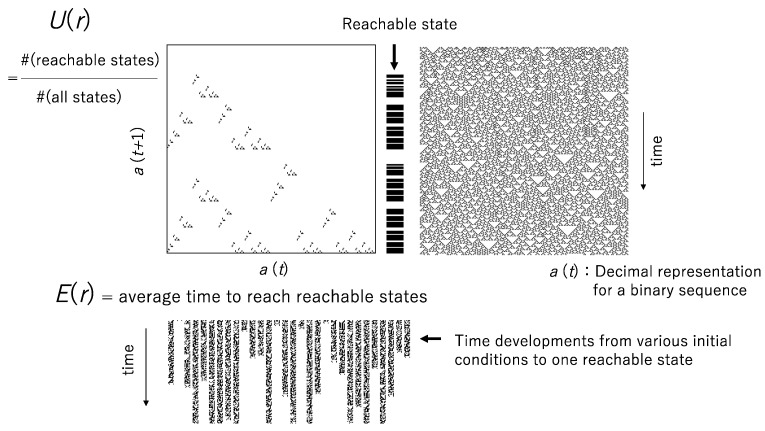
The computational universality and computational efficiency. The cardinality of a set of *a*(*t* + 1) in a return map represents the computational universality, *U*(*r*). The computational efficiency *E*(*r*) is defined by the average time to reach reachable states.

Figure 1 shows a graphical explanation for the computational universality *U*(*r*) and the computational efficiency *E*(*r*). The pattern generated by R18 is shown in Figure 1 right above, and the return map *a*(*t* + 1) plotted against *a*(*t*) is shown in Figure 1 left above, where *a*(*t*) is the decimal expression for a binary sequence. Since *a*(*t* + 1) is calculated for any *a*(*t*) in [0.0, 1.0], a set of *a*(*t* + 1) represents the computational universality. The computational efficiency is obtained from the average time to the reachable states, where the time to a reachable state is obtained from the average of time from all possible initial states to the reachable state, as shown in Figure 1 below.

**Figure 2:**
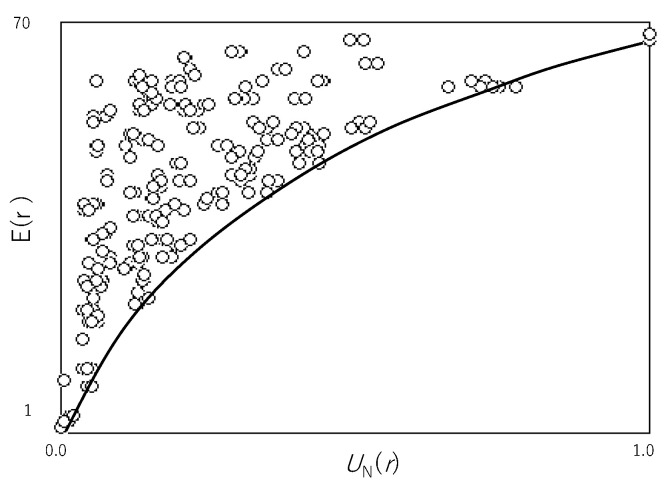
*E*(*r*) plotted against *U*_N_(*r*) for all rules in ECA. Each circle represents a coordinate (*U*_N_(*r*), *E*(*r*)) for each transition rule *r*. The solid curve shows the trade-off between the computational universality and computational efficiency. The parameter is set by the following: *n* = 8, *T* = 7, and *T_θ_* = 70.

Figure 2 shows *E*(*r*) plotted against *U*_N_(*r*) for all rules in ECA. Since *E*(*r*) reveals the average time to reachable states, the smaller *E*(*r*) is, the more efficient *E*(*r*) is. Thus, the minimal point of *E*(*r*) for each computational universality reveals the maximal efficiency for each computational universality. This maximal efficiency is why the solid line representing the lower margin of a cloud of (*U*_N_(*r*), *E*(*r*)) shows the relationship between the computational universality and efficiency. The greater the universality is, the less the efficiency is. It is clear that the solid line shows the trade-off between the computational universality and efficiency.

As mentioned before, the trade-off shown in Figure 2 is obtained by ECA implemented by synchronous updating. If the transition is updated in asynchronous fashion, then what happens with respect to the trade-off between the computational universality and efficiency is discussed below.

## 3. The Trade-Off Breaking by Asynchronous Updating

Asynchronous updating in CA can be implemented using various approaches. One approach is to define the order of updating as defined in the form of bijection from a set of cell sites to the order of updating [35,36,42]. Here, we implement asynchronous updating by introducing the probability variable *p* ∈ [0.0, 1.0] [37,38,39,40]. The transition rule is adapted to each cell with the probability, such that

*a_i_^t^*^+1^ = *f_r_*(*a_i_*_−1_*^t^*, *a_i_^t^*, *a_i+_*_1_*^t^*) with 1 − *p*; = *a_i_^t^* with *p*. (11)

**Figure 3:**
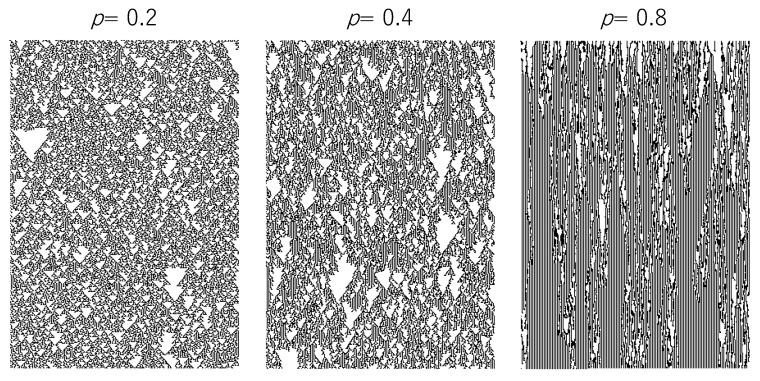
Time development of asynchronous ECA with the probability. The horizontal and vertical lines represent space and time, respectively. The dot and blank represent a state of a cell, 1 and 0, respectively. The transition rule of ECA is R22.

Figure 3 shows the time development of the ECA with the probability, where the transition rule is R22. Since the probability *p* implies the probability of which the transition rule is not applied to a cell, the time development with a small *p* mimics the time development of synchronous ECA.

We estimate *E*(*r*) and *U*_N_(*r*) for asynchronous ECA with the probability, compared to the trade-off between *E*(*r*) and *U*_N_(*r*) in synchronous ECA. For the sake of comparison, the lower margin of the distribution of (*U*_N_(*r*), *E*(*r*)) obtained for synchronous ECA is expressed as a monotonous increasing step function. The interval [0.0, 1.0] is divided into *m* subintervals. The *k*th subinterval, Int*_k_*, is [(*k* − 1)∗1.0/*m*, *k*∗1.0/*m*]. In each subinterval,

*E*_SUB-MIN_(*k*) = min{*E*(*r*) | *U*_N_(*r*) ∈ Int*_k_*}, if there is an element *U*_N_(*r*) exists; = max{*E*(*r*) | *r* = 0, …, 255}, otherwise. (12)

and the monotonous increasing step function, *E*_MIN_(*k*) is defined by

*E*_MIN_(*k*) = min{*E*_SUB-MIN_(*s*) | *k* ≤ *s* ≤ *m*} (13)

**Figure 4:**
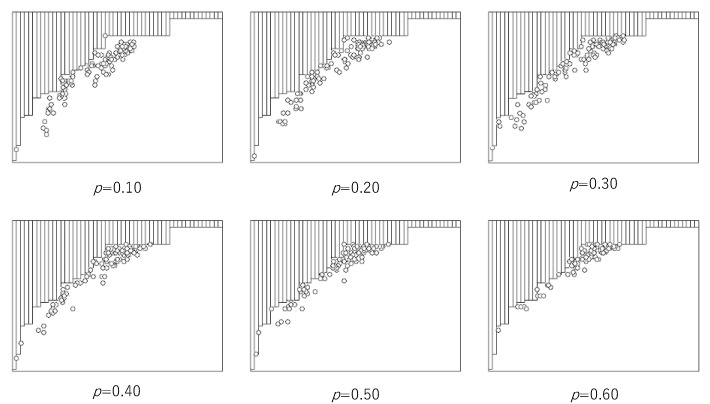
Breaking the trade-off between the computational universality and efficiency by asynchronous ECA with the probability, *p*. Pairs, (*U*^A^_N_(*r*), *E*^A^(*r*)) breaking the trade-off is represented by circles. In each diagram, the horizontal and vertical lines represent the computational universality and efficiency, respectively.

Figure 4 shows the breaking trade-off between the computational universality and efficiency by asynchronous ECA with the probability *p*, where the lower margin of the distribution of (*U*_N_(*r*), *E*(*r*)) is expressed as Equation (13), and *m* = 52. In each graph, the horizontal and vertical lines are the same as those in Figure 2. In Figure 4, all pairs of (*U*_N_(*r*), *E*(*r*)) obtained by synchronous updating are hidden by bars above the increasing step function. The pairs of (*U*^A^_N_(*r*), *E*^A^(*r*)) obtained by asynchronous updating with the probability *p* are represented by circles below the increasing step function. It is easy to see that asynchronous updating with a wide region of *p* entails breaking the trade-off.

**Figure 5:**
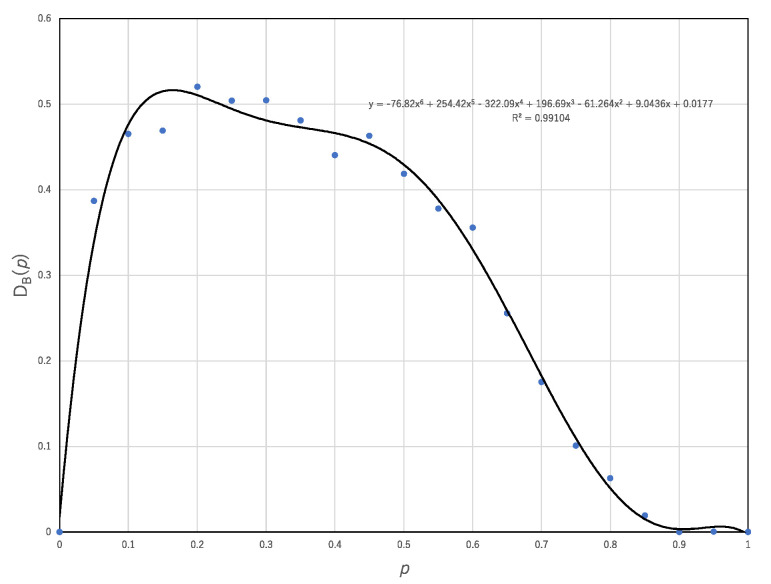
Breaking degree of the trade-off between the computational universality and efficiency plotted against the probability by which asynchronous updating is implemented.

Figure 5 shows the breaking degree of the trade-off plotted against the probability, *p*. As shown in Figure 4, one can count the number of (*U*^A^_N_(*r*), *E*^A^(*r*)) breaking the trade-off obtained by synchronous ECA, which is represented by circles below the lower margin of the distribution of (*U*_N_(*r*), *E*(*r*)) with synchronous updating. The breaking degree, *D*_B_(*p*) for ECA asynchronously updated with the probability, *p*, is defined by

*D*_B_(*p*) = #{*E*^A^(*r*) | *E*^A^(*r*) < *E*_MIN_(*k*), *k* = 1, …, *m*}/256, (14)

where the number 256 represents the number of all ECAs. Figure 5 shows that approximately 50% of transition rules break the trade-off. This result implies that asynchronous updating can reach the reachable states more quickly than synchronous updating so far as the computational universality of asynchronous updating is the same as that of synchronous updating.

The next question arises regarding how asynchronous updating can break the trade-off between the computational universality and efficiency. It is strongly relevant for the universal criticality resulting from asynchronous updating. As mentioned before, the perspective of the phase transition and/or the edge of chaos is obtained in the framework of synchronous updating. We previously proposed the asynchronously updated automata implemented by a bijective map from the address of the cell to the order of updating (order-oriented asynchronous updating) [35,36]. Even if a transition rule shows either order (class 1, 2) or chaos (class 3) in synchronous updating, the same transition rule operated by the order-oriented asynchronous updating shows cluster-like patterns that mix the order with chaos (class 4). Since the cluster-like patterns are characterized by the power law in time development, it can be considered that asynchronous updating entails universality that is independent of the structure of a transition rule.

Asynchronous updating can mix with various transition rules. Even if a transition (0, 0, 1) → 1 is defined, if the transition rule is not applied to a cell, then the state of a middle cell in a triplet is not changed, which implies (0, 0, 1) → 0. This results in an apparent change in the transition rule from R18 to R16 since *d*_1_ = 1 is replaced by *d*_1_ = 0. Here, the transition rule approximated for a pair of binary sequences, (*a*_1_*^t^*, *a*_2_*^t^*, …, *a_n_^t^*) and (*a*_1_*^t^*^+1^, *a*_2_*^t^*^+1^, …, *a_n_^t^*^+1^) is called an apparent rule. For R18, one can see various apparent changes in the transition rule, as shown in Table 1. If *p* = 0.0, then the apparent rule is the same as the transition rule, R18. The larger *p* is, the more apparent the change in *d_s_* is. The lowest row shows the case of *p* = 1.0, which leads to the apparent rule being R204. In 0 < *p* < 1, time development can be interpreted to be generated by various apparent rules showing classes 1, 2 and 3 in time and space. That is why a cluster-like pattern is generated by mixing with up class 1, 2 and 3 transitions.

Mixing with classes 1, 2 and 3 results from asynchronous updating; thus, it can ubiquitously generate cluster-like patterns and/or critical behavior. Since such behaviors correspond to the edge of chaos or the critical state in the phase transition, they can reveal the balance of the computational universality and efficiency. Additionally, these behaviors can entail breaking the trade-off between the universality and efficiency.

To manifest how asynchronous updating breaks the trade-off between the computational universality and efficiency, we approximate the transition of configurations by the asynchronous updating of a single rule by the synchronous updating of multiple rules. Then, we estimate how the number of multiple rules and segmentations can contribute to breaking the trade-off.

Given a binary sequence, the asynchronous updating of a single transition rule defined by d^*^_s_ with *s* = 0, 1 …, 7, is adapted to the binary sequence. It results in a pair of binary sequence such as

(*a*_1_*^t^*, *a*_2_*^t^*, …, *a_n_^t^*); (*a*_1_*^t^*^+1^, *a*_2_*^t^*^+1^, …, *a_n_^t^*^+1^). (15)

For this pair, a binary sequence (*a*_1_*^t^*, *a*_2_*^t^*, …, *a_n_^t^*) is divided into multiple segments,

{(1, *a*_1_*^t^*), (2, *a*_2_*^t^*), …, (*m*, *a_m_^t^*)}, {(*m*+1, *a_m_*_+1_*^t^*), (*m*+2, *a_m_*_+2_*^t^*), …, (*h*, *a_h_^t^*)}, …, {…, (*n*, *a_n_^t^*)}, (16)

where in a segment {(*u*, *a_u_^t^*), (*u*+1, *a_u_*_+1_*^t^*), …, (*w*, *a_w_^t^*)}, for any *s* ∈ {0, …, 7}, if there exists (*a_k_*_−1_*^t^*, *a_k_^t^*, *a_k_*_+1_*^t^*) ∈ **B**^3^ such that *s* = 4*a_k_*_−1_*^t^* + 2*a_k_^t^* + *a_k_*_+1_*^t^*, *k* ∈ {*u*, *u* + 1, …, *w*},

*d_s_* = *a_k_^t^*^+1^ (17)

and otherwise,

*d_s_* = d^*^_s_. (18)

This implies that for each segment, one can uniquely determine the corresponding rule defined by *d_s_* with *s* = 0, 1 …, 7 and that a sequence, (*a_u_^t^*, *a_u_*_+1_*^t^*, …, *a_w_^t^*); (*a_u_^t^*^+1^, *a_u_*_+1_*^t^*^+1^, …, *a_w_^t^*^+1^) can be interpreted as a transition generated by the synchronous updating of a single transition rule. Thus, segmentation (10) implies the approximation of which each segment can be generated by a single transition rule and a whole sequence can be synchronously generated by multiple transition rules.

**Figure 6:**
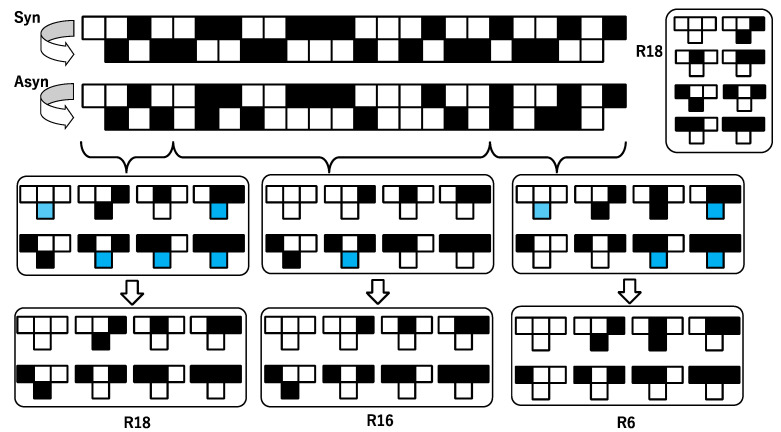
Schematic diagram of the approximation for a pair of binary sequences generated by the asynchronous updating of a single rule (R18) approximated by the synchronous updating of multiple rules (R18 + R16 + R6). States 1 and 0 in a cell are represented by filled and blank squares, respectively. The symbols “Syn” and “Asyn” represent synchronous and asynchronous updating, respectively. See text for the detailed discussion.

Figure 6 shows an example of the approximation by the synchronous updating of multiple rules. The top pair of binary sequences with “Syn” is a transition generated by the synchronous updating of the rule, R18. The top second pair with “Asyn” is a transition generated by asynchronous updating with a certain probability. Please note that due to the probability, there are some cells where *a_k_^t^*^+1^ = *a_k_^t^*. In Figure 6, a transition generated by asynchronous updating is divided into three segments. Algorithmically, the segmentation is implemented from left to right. From the first cell, one can determine *d*_1_ = *a*_1_*^t^*^+1^ = 1, and then *d*_2_ = *a*_2_*^t^*^+1^ = 0, *d*_4_ = *a*_3_*^t^*^+1^ = 1. At the fourth cell at *t*+1, one obtains *d*_1_ = *a*_4_*^t^*^+1^ = 0 and that conflicts with *d*_1_ = (*a*_1_*^t^*^+1^) = 1. That is why the first segment is terminated by the third cell at *t*+1, which is expressed as {(0, *a*_0_*^t^*), (1, *a*_1_*^t^*), (2, *a*_2_*^t^*), (3, *a*_3_*^t^*)}. For a transition rule, only *d*_1_, *d*_2_ and *d*_4_ are determined, and *d*_0_, *d*_3_, *d*_5_, *d*_6_ and *d*_7_ are not determined in that segment, {(0, *a*_0_*^t^*), (1, *a*_1_*^t^*), (2, *a*_2_*^t^*), (3, *a*_3_*^t^*)}. The undetermined value *d_s_* for a transition rule is represented by the blue cell in Figure 6. By definition (18), the undetermined *d_s_* is substituted by d^*^_s_, which is defined by R18 in Figure 6. Thus, for the first segment in Figure 6, one can obtain R18. Similarly, it results in three segments, and the second and third segments are approximated by R16 and R6, respectively.

**Figure 7:**
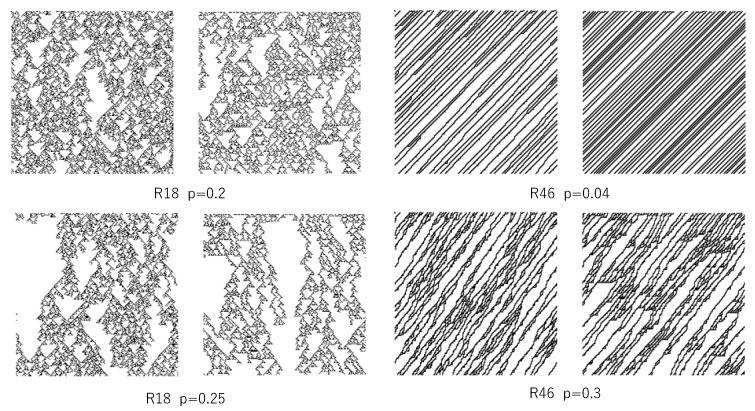
Four examples of the approximation for the transition of asynchronous CA by the transition of synchronous multiple CAs. In each diagram pair, the left diagram shows the time development by asynchronous CA, and the right diagram shows that by synchronous multiple CAs. The transition rule number and the probability defined by Equation (6) are shown at the bottom middle of each pair. The dot represents a cell whose state is 1, and the blank represents a cell whose state is 0.

Figure 7 shows some examples of a pair of time developments by the asynchronous updating of a single rule and the corresponding time development emulated by the synchronous updating of multiple rules. In a pair of time developments, left above, the left diagram represents the time development of the asynchronous updating of R18 with a probability of 0.2. For this asynchronous CA, given 10^4^ cells whose values are randomly set, the segmentation procedure is run. This process results in *N*_1_ segments and *N*_2_ transition rules. By using *N*_1_ segments and *N*_2_ transition rules, the approximated time development is emulated. First, at each cell, it is probably determined whether the segment is cut or not, with the probability of *N*_1_/10^4^ (segmentation process). Second, a transition rule randomly chosen from *N*_2_ transition rules is applied to each segment, and the state of cells is updated (update process). Both segmentation and update processes are performed for each time step, which leads to time development, as shown in the right diagram of each pair. Clearly, the synchronous updating of multiple rules can emulate the time development of asynchronous updating of a single rule. In other words, the behavior of asynchronous CA can be estimated by synchronous CA with multiple rules.

**Figure 8:**
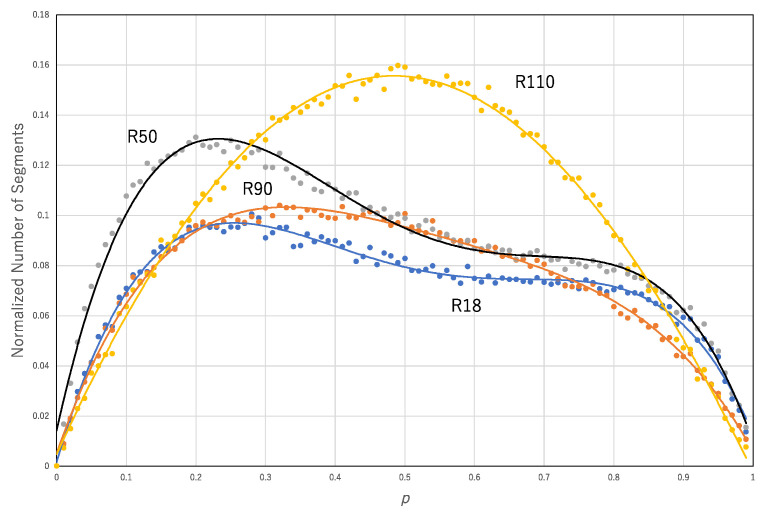
Normalized number of segments (*N*_1_/10^4^) against the probability *p* in the approximation of asynchronous updating by the synchronous updating of multiple rules. Each curve represents a single transition rule, R110 (yellow), R50 (gray), R90 (orange) and R18 (blue).

Given *p*, a transition rule, and 10^4^ cells whose states are randomly determined, the asynchronous updating of the transition rule with probability *p* is applied to 10^4^ cells. For a pair of binary sequences of the initial configuration and results of application of the transition rule, the segmentation process is applied. This process results in a pair of the number of rules and the number of segments. Figure 8 shows the normalized number of segments (*N*_1_/10^4^) against *p* for some of the asynchronous updating of the transition rules, R110, R50, R90 and R18. The data for each transition rule are approximated by a polynomial function: for R110, *y* = 0.1081*x*^4^ − 0.1643*x*^3^ − 0.5596*x*^2^ + 0.6087 *x* + 0.0048, R^2^ = 0.99594; for R50, *y* = −2.7514*x*^4^ + 5.9832*x*^3^ − 4.492*x*^2^ + 1.2555*x* + 0.0143, R^2^ = 0.98422; for R90, *y* = −1.1571*x*^4^ + 2.6079*x*^3^ − 2.2464*x*^2^ + 0.7964 *x* + 0.0051, R^2^ = 0.99293; and for R18, *y* = −2.0516*x*^4^ + 4.4103*x*^3^ − 3.3139*x*^2^ + 0.9666*x* + 0.0016, R^2^ = 0.98778.

**Figure 9:**
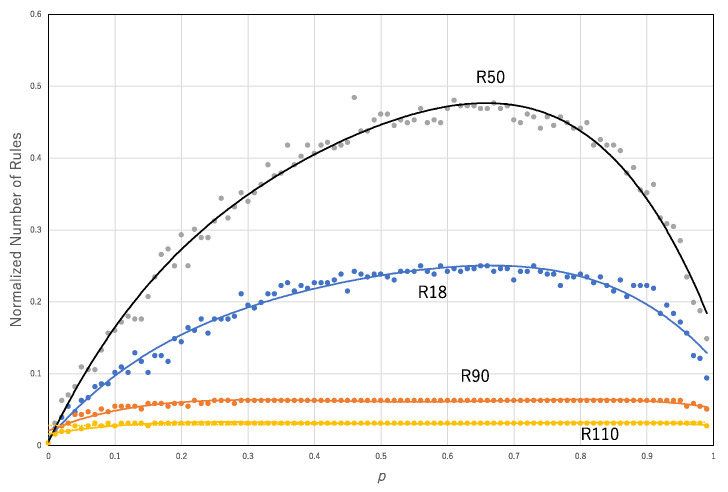
Normalized number of transition rules (*N*_2_/256) against the probability *p* in the approximation of asynchronous updating by the synchronous updating of multiple rules. Each curve represents a single transition rule, R110 (yellow), R50 (gray), R90 (orange) and R18 (blue).

For the same approximation, Figure 9 shows the normalized number of rules (*N*_2_/256) against *p* for each transition rule. The data for each transition rule are approximated by a polynomial function: for R110, *y* = −0.3159*x*^4^ + 0.706*x*^3^ − 0.5401*x*^2^ + 0.1619; for R50, *y* = −2.7529*x*^4^ + 4.7656*x*^3^ − 3.7832*x*^2^ + 1.9264*x* + 0.0054, R^2^ = 0.98541; for R90, *y* = −0.6852*x*^4^ + 1.5296*x*^3^ − 1.1948*x*^2^ + 0.3817*x* + 0.0208, R^2^ = 0.90307; and for R18, *y* = −2.0516*x*^4^ + 4.4103*x*^3^ − 3.3139*x*^2^ + 0.9666*x* + 0.0016, R^2^ = 0.98778.

**Figure 10:**
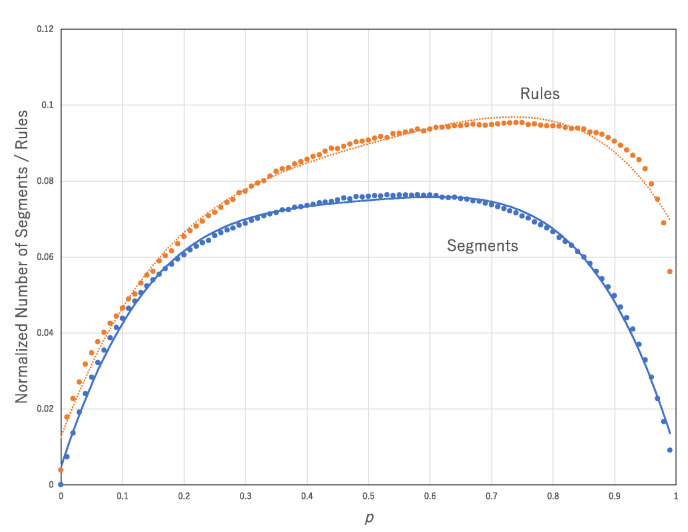
Normalized number of segments and normalized number of transition rules against *p* averaged over all 256 transition rules. These parameters are obtained from the approximation of the asynchronous updating of a single rule approximated by the synchronous updating of multiple rules.

Both curves, the normalized number of segments and the normalized number of transition rules against *p* show convex functions for each transition rule (Figure 8 and Figure 9). Figure 10 shows the normalized number of segments against *p* and normalized number of transition rules against *p* averaged over all 256 transition rules. The former and latter graphs are approximated by *y* = −0.9837*x*^4^ + 1.9304*x*^3^ − 1.4477*x*^2^ + 0.5047*x* + 0.0047, R^2^ = 0.99594, and *y* = −0.6488*x*^4^ + 1.333*x*^3^ − 1.0644*x*^2^ + 0.4345*x* + 0.0125, R^2^ = 0.98811, respectively. The normalized number of rules and segments in the approximation might contribute to an increase in the computational efficiency since it can increase the diversity of the configurations. However, it is not necessary that the diversity of configurations is implemented by the diversity of rules. R90 and R150 can compute any configurations if the corresponding initial condition is prepared.

Therefore, if the asynchronous updating of R90 (R150) is applied to an initial configuration, then one can obtain not multiple rules but multiple segments in the approximation of synchronous updating. This procedure implies that all segments can be synchronously updated by a single rule, R90 (R150). It is the case that the diversity of configurations can be achieved by a single rule. There are some similar cases with R90 and R150. Those transition rules show chaotic and/or spatially propagating wave patterns referred to as class 3 or 4. These classes are shown by the high value of the number of segmentations divided by the number of rules (represented by #Segments/#Rules) in the approximation. In contrast, if the approximated rules cannot contribute to the diversity, then one obtains many various rules contributing to the diversity. These transition rules show locally stable behavior called class 1 or class 2. In this case, one can see a high value of #Rules/#Segments.

**Figure 11:**
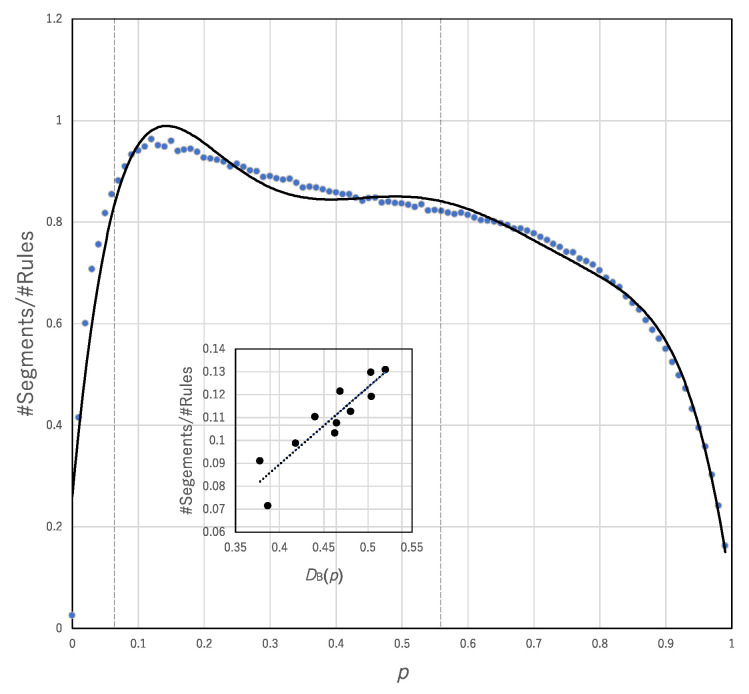
#Segments/#Rules plotted against *p*. The data are approximated by *y* = −126.41*x*^6^ + 403.31*x*^5^ − 506.65*x*^4^ + 314.36*x*^3^ − 98.944*x*^2^ + 14.135*x* + 0.2609, R^2^ = 0.96549. The inner graph shows the linear regression between *D*_B_(*p*) and #Segments/#Rules.

We estimate whether #Segments/#Rules or #Rules/#Segments can influence the break of the trade-off between the computational universality and computational efficiency. Figure 11 shows #Segments/#Rules plotted against *p*. The range 0.5 < *p* < 5.5 surrounded by broken lines represents the range in which the trade-off is broken. In that range, the coefficient of determination between #Segments/#Rules and the degree of break of the trade-off, *D*_B_(*p*), is very high (R^2^ = 0.82076), whereas the correlation between #Rules/#Segments and *D*_B_(*p*) is very low (R^2^ = 0.56706). This finding suggests that the diversity resulting from a smaller number of transition rules (i.e., class 3 or 4-like behavior) contributes to breaking the trade-off compared with the diversity resulting from a large number of transition rules (i.e., class 1 or 2-like behavior). In other words, although there are both effects of generalists with high #Segments/#Rules and specialists with high #Rules/#Segment in asynchronous updating, only effect from generalists can contribute to break of the trade-off.

## 4. Conclusions

While natural and bioinspired computing seems to be different from computations based on the Turing machine, there was no plan to extend the notion of the computational universality and efficiency beyond the Turing machine. On the other hand, although it is known that the critical state or computation at the edge of chaos can be used for an adequate solution but not for optimal solution, there have been few studies that bridge the critical state with the balancing of the universality and efficiency in computation. To connect these two issues, one should quantify the universality and efficiency in a certain computational system that can emulate natural and bioinspired computing.

To solve this problem, we quantify computational universality and efficiency in cellular automata and show the trade-off between universality and efficiency in synchronous cellular automata. Since asynchronous updating is much more adequate in natural and biocomputing, we estimate how the relationship between the computational universality and efficiency is influenced by replacing synchronous updating with asynchronous updating. We define ECA asynchronously updated by introducing the probability of relaxation and compare the relation between the universality and efficiency in synchronous ECA with the relation in asynchronous ECA. This comparison leads to the finding that asynchronous ECA breaks the trade-off found in synchronous ECA. Via the same universality, the efficiency in asynchronous ECA is much more than that in synchronous ECA.

What is the main cause to break the trade-off by asynchronous updating? To answer this question, we emulate patterns that are generated by the asynchronous updating of a single transition by the synchronous updating of multiple transition rules. Through this emulation, one can estimate the potential diversity of asynchronous updating with respect to the number of segments and the number rules. Our analysis suggests that asynchronous updating contributes to increasing the segmentation rather than the transition rule, which has the potential to generate various configurations, which can play an essential role in breaking the trade-off between the universality and efficiency.

## Figures and Tables

**Table 1:** The apparent change in a transition rule originated from R18. If a transition rule R18 is not adapted to cells with some probability, then some *d_s_*s are changed, and the apparent rule number is changed. The columns *d_s_* represent the apparent transition due to the asynchronous updating with the probability. The column AR represents the apparent rule number and their corresponding classes.

*d*_0_	*d*_1_	*d*_2_	*d*_3_	*d*_4_	*d*_5_	*d*_6_	*d*_7_	AR	Class
000	001	010	011	100	101	110	111		
0	1	0	0	1	0	0	0	R18	3
0	0	0	0	0	0	0	0	R0	1
0	1	0	1	1	0	1	0	R90	3
0	1	0	0	1	0	0	1	R146	3
0	0	0	0	0	0	0	1	R128	1
0	0	1	1	0	0	1	0	R76	2
0	0	1	1	0	0	1	1	R204	2
